# Configuration of the Geometric State of Railway Tracks in the Sustainability Development of Electrified Traction Systems

**DOI:** 10.3390/s23052817

**Published:** 2023-03-04

**Authors:** Arkadiusz Kampczyk, Katarzyna Rombalska

**Affiliations:** Department of Engineering Surveying and Civil Engineering, Faculty of Geo-Data Science, Geodesy, and Environmental Engineering, AGH University of Science and Technology, al. A. Mickiewicza 30, 30-059 Krakow, Poland

**Keywords:** catenary, return network, electrified traction system, railway track geometry, estimates of railway track condition, electric traction, monitoring, surveying, diagnostics, measuring instruments, sustainability development

## Abstract

The state-space interface of the railway track (track) geometry system with an electrified traction system (ETS) constitutes the geometric configuration that is utilised in this study. Importantly, driving comfort, smooth operation (smooth running), and compliance with the ETS are the desired aims. Direct measurement methods were used in the interaction with the system, especially in regard to the fixed-point, visual, and expert methods. In particular, track-recording trolleys were used. The subjects belonging to the insulated instruments also included the integration of certain methods, such as in the brainstorming, mind mapping, system approach, heuristic, failure mode and effect analysis, and system failure mode effects analysis methods. These were based on a case study and are representative of three real objects, i.e., electrified railway lines, direct current (DC), and scientific research objects (which specifically cover five research objects). The aim of the scientific research work is to increase the interoperability of the railway track geometric state configurations in regard to the sustainability development of the ETS. The results of this work confirmed their validity. By ensuring that the six-parameter defectiveness *D_6_* was defined and implemented, the *D_6_* parameter of the railway track condition was first estimated. The new approach reinforces the improvement in preventive maintenance and reductions in corrective maintenance; moreover, it is an innovative supplement to the existing direct measurement method in the configuration of the geometric condition of railway tracks and in the sustainability development of the ETS via interacting with the indirect measurement method.

## 1. Introduction

The state-space interface of the railway track (track) geometry system with the electrified traction system (ETS) constitutes the geometric configuration that is utilised in this study. Specifically, ensuring driving comfort, smooth operation (smooth running), and compliance with the ETS are the desired aims of this study. Leading in wheel/rail and pantograph/catenary interaction and in safety, the railway sector with its traction system, along with other elements, constitutes a fully functioning railway infrastructure. Indeed, the facilities for the conversion and distribution of electricity for the traction power supply—such as traction substations, power cables between traction substations and contact wires, catenary with supporting structures, and the third rail (current rail) with supporting structures [1]—are part of the railway infrastructure, provided they form part of a railway line, station siding (railway siding), or other railway roads (or that are intended for their management, the operation of the carriage, the delivery of passengers or goods, or their maintenance [1]). Vithanage et al. in [2] conclude that maintenance, which is critical for safe, reliable, quality, and cost-effective service, plays a dominant role in the railway industry. Márquez in [3], when dealing with the subject of the maintenance management framework (MMF), provides a perspective on traffic management, particularly in regard to maintenance actions. In turn, Konowrocki and Chojnacki in [4] state that issues such as the safety and reliability of rail vehicle traffic are being improved and, thus, require consideration during design. The subject of the railway maintenance system is also addressed by Song and Schnieder in [5], whereby they recognise that to achieve high levels of maintenance, an appropriate strategy must be employed. In addition, they emphasise that it is important to carry out a so-called performance evaluation of the strategy before putting it into practice. The problems of investigating the subject of pantograph–catenary correlations in railway tracks were addressed by the authors of the publications [6,7,8,9,10,11,12]. Attention was drawn to a new methodology for studying pantograph–catenary dynamics in curved railway tracks [6] and the distribution of magnetic and electric fields [7]. In addition, the studied medium-voltage direct current railway traction power systems (MVDC RTPS) [8], and the different types of configurations of railway traction electrical power systems [9], and the presented measures to reduce energy consumption are based on urban railways (urban rail) [10]. Douglas et al. in [11] found that the achievement of savings varies by route, which is then followed by vehicle and service characteristics. Meanwhile, Novak et al. in [12] focus their scientific research work specifically on electric traction substation energy flows and energy consumption. The efficiency of rail transport infrastructure (rail transportation infrastructure), particularly in the railway sector, includes the configuration of the geometric state of railway tracks in the sustainability development of the ETS. With regard to the type of railway track construction, a distinction should be made between a jointed railway track (jointed rail, jointed track, and JRT) and a continuous welded rail track (CWR track) [13,14,15]. Atapin et al. in [16] recognise that the CWR track has great advantages in respect of low-cost maintenance, environmental influence, and ride comfort. They also emphasise that irregularities caused an increase in temperature stress in the rails during both construction and maintenance at the same time and during the particular conditions of the influence of train loads, natural factors, and of climatic factors. The interaction of factors (e.g., temperature spikes, creeping of the rails, incorrect conditions, and operational impacts (exploitation impacts)) can lead to damage or a deformation of the track’s construction, thereby endangering operational safety (exploitation safety). The issue of CWR track stability and its monitoring is also discussed in the scientific research publication by Németh and Fischer in [17], particularly in regard to focusing on the investigation of glued insulated rail joints that are applied to CWR tracks. Then, finding a reflection in these issues, such as in the investigation of the horizontal track geometry regarding geogrid reinforcement under ballast, was realised by Fischer in [18]. In addition, the maintenance reliability of railway curves, using their design parameters, was realised by Kurhan et al. in [19]. At the same time, Andrade and Teixeira emphasise in [20] that the degradation of ‘rail track geometry’ is responsible for the largest share of railway infrastructure maintenance costs. Chen et al. in [21] state that ‘track irregularity, i.e., track deformation, is one of the most important factors that cause safety problems and further track deterioration’, noting the importance of carrying out an accurate measurement of the track geometry.

Rail transportation, specifically railway transport, is a fundamental part of the wider mobility strategy; it plays an important role in the sustainable development goals (SDGs). Loo and Comtois in [22] state that the railway in the ‘context of comprehensive sustainability’ is represented by sustainable development, social and economic equity, and community liveability.

Kostrzewski and Chudzikiewicz in [23], when conducting scientific research work on the subject of the rail vehicle and rail track monitoring system (a key part of transport sustainable development), underline the need to ensure the safety of the integrated transport system. The mode of transport that seems to be the least harmful to the natural environment is railroad transport. Thus, Wangai et al. in [24] pay particular attention to harmonising the interaction between ‘society, economic demands, technological development, and regulation’ in their new methodology approach.

At the same time, Guliy et al. in [25] conclude that the development of railway transport is sustainable, or at least close to sustainable. Açikbas and Söylemez in [26] confirm that rail systems are well-known for their energy efficiency. The energy resources used around the world in mass rail transit systems is predominantly electricity. It is, therefore, incredibly important that efficiency is increased, as this is also an essential requirement for sustainable development. Thus, Faranda and Leva in [27], when dealing with the energetic sustainable development of railway stations, note that, in the near future, one important solution could be the application of a photovoltaic power generator in railway stations. At the same time, Liu et al. in [28] conclude that the bad state of catenaries is able to directly influence the power supply safety of traction power systems. The traction power supply system includes the following:

- A set of generating equipment and facilities;
- A set of processing equipment and installations;
- Transmission and distribution systems.

**Figure 1:**
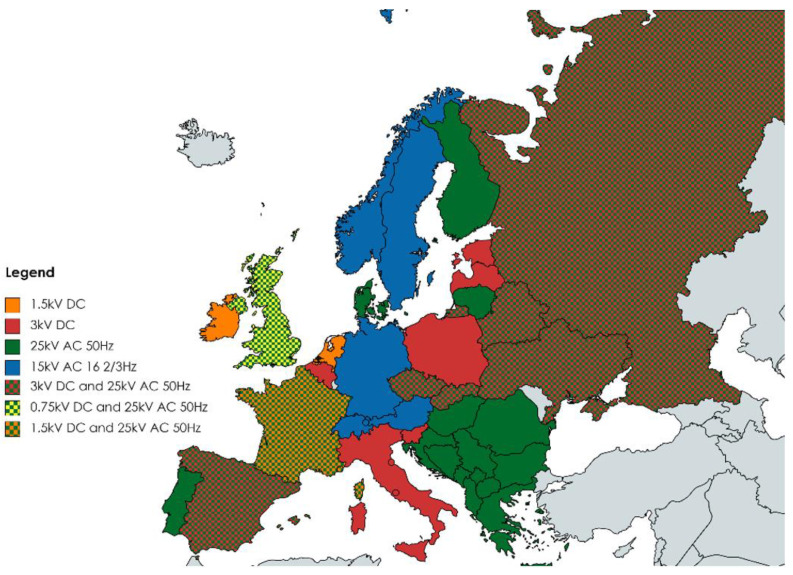
Rail transport electro-energy systems in European countries, the general condition.

Through the above, electricity is provided to the catenary. In turn, this system then powers the electric traction vehicles. The kinds and types of railroad electric traction power systems in Europe are shown in Figure 1. The characteristics of the types of power system are determined by the types of voltage:

- Direct current—denoted as DC;
- Alternating current—denoted as AC.

Then, the system is characterised by the value and frequency (possibly the number of phases) of the voltage in the catenary.

Abrahamsson et al. in [29] recognise the useful features of interaction in regard to the public power grids towards the grids supplying power to the railways. Additionally, what is particularly important is that they emphasise that the converters are especially favourable, regardless of whether the overhead contact lines are of a DC type or an AC type.

It should be particularly noted that the subject of scientific research work, regarding the configuration of the geometric state of railway tracks in the sustainability development of the ETS, is important, especially since steel is the basis of the structural materials that are primarily used in rail transportation. The task of the railway track is to primarily ensure the safe movement of traction vehicles on the designated railroad, especially since the catenary covers the contact line and return network.

**Figure 2:**
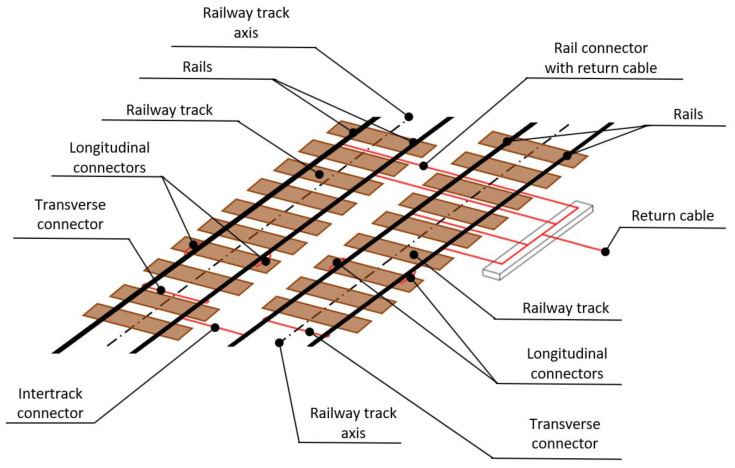
Connection scheme in the return network.

At the same time, the contact line should be compatible with the geometry of the railway track. A return catenary, on the other hand, includes a segment of circuit that is connected to a return pole in a traction substation on one side and to a vehicle contact wire on the other. This is also how the return circuit includes return conductors and running rails. Thus, railway tracks perform another particularly important function in rail transportation, i.e., they are the conductors of the return current. Railway tracks as conductors carry currents from traction vehicle engines and short-circuit currents to the traction substation. They also have applications in terms of the control command and signalling (CCS) system. A schematic diagram of the constituent elements of the return network is shown in Figure 2, whereby appropriate connections between rails and tracks and between railway tracks—which is where the rail bonding is used—are required.

Fundamental to monitoring the condition of railway infrastructure are the acts of surveying and diagnostic monitoring, which is an issue addressed by Kampczyk and Dybeł in [30]. They addressed the subject of integrating and surveying railway special grid pins with terrestrial laser scanning targets for monitoring rail transport infrastructure. This is a task that is a global responsibility, especially in the discipline of civil engineering, surveying/geodesy, and transport. It also has applications in monitoring the state-space interface of the railway tracks’ geometry system, with the electrified traction system as the geometric configuration. The aforementioned play a key role in terms of understanding how they can affect not only the real conditions of both the space interface but also the environmental conditions. At the same time, they contribute to the proper operation of the power networks.

Surveying work and diagnostic work are essential parts of dealing with any structural object or engineering infrastructure object. This is the case during design and measurement, during the construction process, and in their use (operational, exploitation). At the same time, as Mrówczyńska et al. in [31] emphasise, this type of work is conditioned by the nature of the site and the specific field conditions, requiring the adaptation of measurement methods and of data-processing methods. At the same time, the common denominator is the need to combine experimental and theoretical research [32]. This is confirmed by the results of the scientific research work carried out in the configuration of the geometric state of railway tracks in regard to the sustainability development of the ETS.

The main objective of the scientific research work is to increase the interoperability of railway tracks’ geometric state configuration in the sustainability development of the ETS. Requiring an analysis and evaluation (assessment, estimates) of the ‘changes’ in the values of the track condition indicator *J* and the five-parameter defectiveness *D_5_* is common to estimate the railway track condition, which—at the same time—provides the basis for making decisions on maintenance interventions.

Scientific research work (in the territory of Poland) was carried out on three real objects (railway lines, object Nos. R_143_, R_144_, and R_161_) covering five research objects (scientific research object was carried out on five railway tracks, object Nos. R_143_1_1_ and R_143_1_2_, R_143_1_2_, R_144_1_, R_161_1_, and R_161_2_), characterised by different geometric features in plan and profile and exploitation elements of transport engineering. The scientific research works were conducted from 2019, obtaining four time horizons (*M_1_* ÷ *M_4_*):

- Object Nos. R_143_1_1_ and R_143_1_2_—Railway track No. 1: *M_1_* ÷ *M_4_*;
- Object No. R_143_1_2_—Railway track No. 2: *M_1_* ÷ *M_4_*;
- Object No. R_144_1_—Railway track No. 1: *M_1_* ÷ *M_4_*.

Additionally, works were conducted from 2021, obtaining two time horizons (*M_3_* ÷ *M_4_*):

- Object No. R_161_1_—Railway track No. 1: *M_3_* ÷ *M_4_*;
- Object No. R_161_2_—Railway track No. 2: *M_3_* ÷ *M_4_*_._

The results of the scientific research works carried out were supplemented with the values of the state of the geometrics of the contact line, as represented by the suspension height of the contact line (suspension height and suspension height of the contact wire) and the contact line stagger (offset, offset of the contact line). The identification of real objects as they are introduced in the publication have their anonymity preserved, such that no direct identification can be made for a given construction object. At the same time, the scientific research work was completed on real objects, which function in everyday life, and the data was adequately presented. As such, a publication of great interest was constructed, with innovation and depth, both for other readers and for a range of international researchers and practitioners. The results of the scientific research works confirmed the validity of the parameters. Furthermore, they are well-established definitions and equations, and the implementation of a new parameter for the purposes of estimating the railway track condition (assessments of the track condition), i.e., the six-parameter defectiveness *D_6_*, was confirmed. This new approach, as an innovation, complements the configuration of the geometric state of railway tracks in respect of the sustainability development of the ETS. At the same time, reinforcing the improvement of preventive maintenance and the reduction in corrective maintenance, as an innovative supplement, means that the existing direct measurement method in the configuration of the geometric state of railway tracks in the sustainability development of the ETS is particularly maintained. This also has a corollary in the indirect measurement method. As a result of introducing innovations and contributions to the development of the engineering and technical field—particularly in the discipline of civil engineering, surveying/geodesy, and transport—the implemented innovation enhances the interoperability of the railway tracks’ geometric state configuration in the sustainability development of the ETS.

## 2. Materials and Methods

The arrangement of the scientific research work in this section consists of three essential parts. The first is defined by the railway track geometry, the second is in regard to the electrified traction system, and the third relates to the scientific research and industry-specific methods. Rail transportation infrastructure requires their strong correlation, especially in the configuration of the geometric state of railway tracks in the sustainability development of electrified traction systems. The aim of sustainable development was firmly grounded in European treaties. The agenda for sustainable development by 2030 came with certain SDGs [33], which have given a strong impetus to international challenges. The SDGs have an active role in supporting progress. One of the main objectives of sustainable development is to ‘build resilient infrastructure, promote inclusive and sustainable industrialisation and foster innovation’ (target number 9) [33]. This objective is supported by the ongoing scientific research work into the configuration of the geometric state of railway tracks, specifically in regard to the sustainability development of the ETS rail transport infrastructure.

### 2.1. Railway Track Geometry

The geometry of the railway track is the basis for the design, construction, modernisation, revitalisation, and maintenance of the railway track. Chen et al. in [21] state that the accurate measurement of railway track geometry is a task of fundamental importance to ensure the track quality in both the construction phase and the regular maintenance stage. They also recognise that railway track geometry is characterised by its external and internal geometric parameters. The configuration of the geometric state of railway tracks in the sustainability development of the ETS with respect to monitoring the status of their geometry requires a huge amount of data. This is also recognised by Khosravi et al. in [34], especially since a large volume of data is periodically collected from the railway track to monitor its geometry condition. This massive amount of data, which exhibits the classical characteristics of big data, includes measurements of all the track geometry parameters. These measurements are used to evaluate the geometry condition and to predict the occurrence of geometry defects [34,35]. Increased demand for railway transportation is creating a need for higher train speeds and axle loads. These, in turn, increase the likelihood of track degradation and failures [35]. The basic element supporting the railway surface (permanent way, permanent track, and railway superstructure) is the railway track. Additionally, its geometric layout should ensure the safe movement of rail vehicles.

#### 2.1.1. Architecture of Railway Track Geometric Parameters

The architecture of the geometric parameters of the railway track determines and plays a decisive role in the configuration of the geometric state of railway tracks, and in the sustainability development of electrified traction systems. It has an obligatory place in the creation and maintenance of order regarding the various elements of rail transport infrastructure and its surroundings. Its main representation is in the system of geometric parameters of the railway track, specifically in the interaction of planes:

1. Horizontal (*H*):

- Track gauge (*Gt*);
- Gradient of the track gauge (track gauge gradient, *GGt*);
- Irregularities of the track rails in the horizontal plane (horizontal irregularities, *Ih*).

2. Vertical (*V*):

- Cant (superelevation, *Cant*);
- Twist (track twist, *Tw*);
- Irregularities of the track rails in the vertical plane (vertical irregularities, *Iv*).

Ensuring the safe architecture and exploitation of the railway tracks requires monitoring, using, surveying, and diagnostic work with measuring instruments, especially in regard to using electronic measurement techniques. This is achieved by providing reliable data for the state-space interface of the railway tracks geometric system with the electrified traction system, i.e., the geometric configuration.

#### 2.1.2. Estimates of Railway Track Conditions

The precursors of an in-depth scientific research subject focusing on the estimates of railway track condition issues were conducted by authors Bałuch and Bałuch in [36,37,38,39]. They particularly focused on railway transport (railway transportation) issues. In terms of the subject of permanent ways, the railway tracks geometry that Bałuch and Bałuch, in [36], focused on was in respect of the layouts of geometrical railway tracks. This was conducted whilst also taking into account the durability of the permanent track. Additionally, Bałuch in [37] takes up the subject of synthetic methods for the evaluation of the railway track. Furthermore, Bałuch and Bałuch, in a scientific research paper in [38], highlight that interpreting the results of their observations and measurements is a skill upon which the proper use of the information collected will depend the most. At the same time, they draw attention to the measuring of railway track condition estimates, dividing them into two groups: relative assessment (relative estimates) and absolute assessment (absolute estimates). At the same time, Bałuch in [39], in his analysis to date of the results of geometric measurements of the railway track, enrich these assessments by their calculations of transverse accelerations and their increments, as based on versine and cant measurements.

The division of railway track condition estimates into relative estimates and absolute estimates is shown in [38]. These were also considered (explored) in a later study [40], which was conducted in correspondence with Id-14 (D-75) [41] and [42].

The group of relative assessments includes the five-parameter defectiveness *D_5_*, which is defined by regulations Id-14 (D-75) [42] and complies with the requirements of the Railway Transport Act of 28 March 2003 [1] under European Union directives. The group of relative assessments of the track condition also includes the new six-parameter defectiveness parameter *D*_6_, which is defined and implemented by the authors. The relative assessments of the track condition change with each change in the permissible deviations of the railway track geometry. These assessments express and show the suitability of the railway track for the assumed exploitation conditions.

The five-parameter defectiveness *D_5_* is a relative measure of railway track conditions, varying with the speed of trains. At the same time, it provides an approximate measure of the railway track’s geometric condition. This parameter is calculated on the basis of the number of exceedances of the limits in the range of the selected class of deviations of permissible geometric parameters. With the limit value already defined as *D_i_*, which is the defectivity for each measured parameter, the five-parameter defectiveness *D_5_* defines Equation (1) [42]:

(1) $$D_{5}=1-\left(1-D_{Gt}\right)\left(1-D_{Cant}\right)\left(1-D_{Tw}\right)\left(1-D_{Iv}\right)\left(1-D_{Ih}\right)$$

where *D_Gt_*—track gauge defectiveness; *D_Cant_*—cant defectiveness; *D_Tw_*—twist defectiveness; *D_Iv_*—the defectiveness of vertical irregularities; *D_Ih_*—the defectiveness of horizontal irregularities. 

Based on the relative assessments of the track conditions represented by the five-parameter defectiveness that is defined in regulation Id-14 (D-75) [42], the geometric condition of the railway track can be estimated in relation to the speed of trains.

A new six-parameter defectiveness parameter *D_6_*, which is analogous to *D_5_*, is also a relative measure of railway track conditions that changes with the speed of trains. It depends on the permissible deviations of the various geometric parameters that are applicable to the railway track segments. It provides a fully indicative measure of the railway track’s geometric condition. It is calculated on the basis of the number of exceedances of the limits within the range of the selected class of deviations of the permissible geometric parameters, with the limit value already defined as defectivity *D_i_* for each measured parameter. The six-parameter defectiveness parameter *D_6_*, as opposed to the defectiveness of the five-parameter *D_5_*, takes into account the defectiveness of the width gradient of the track gauge parameter *D_GGt_*, thus remaining in full interaction of the geometric state of the railway track configuration. Until now, the gradient of the track gauge *GGt* as a defectiveness of the track gauge gradient *D_GGt_* was not included in the relative assessment. *GGt* belongs to the sensitive geometrical parameters in railway tracks. Six-parameter defectiveness parameter *D_6_* defines Equation (2):

(2) $$D_{6}=1-\left(1-D_{Gt}\right)\left(1-D_{Cant}\right)\left(1-D_{Tw}\right)\left(1-D_{Iv}\right)\left(1-D_{Ih}\right)\left(1-D_{GGt}\right)$$

where *D_Gt_*, *D_Cant_*, *D_Tw_*, *D_Iv_*, *D_Ih_*—as according to Equation (1); *D_GGt_*—the defectiveness of the track gauge gradient.

When conducting the calculations of the five-parameter defectiveness parameter *D_5_* and the six-parameter defectiveness parameter *D_6_* in the initial stage, the *D_i_* defectiveness calculations were thus made for each measured parameter on the evaluated baseline section (i.e., base section, section, and railway track baseline section). The length of the baseline sections in this scientific research work is set at 100 m as the standard. According to Id-14 (D-75) [42], the base section is the relation of the sum of the lengths of the sections where the permissible deviations exceed the total length of that section. The *D_i_* defectiveness for each measured geometric parameter of the railway track is calculated according to Equation (3) [42]:

(3) $$D_{i}=\frac{n_{p}}{n}$$

where *n_p_*—the number of signal samples exceeding the deviations allowed on the analysed section; *n*—number of signal samples on the analysed section.

The calculated *D_i_* defectiveness values of each measured geometric parameter of the railway track involving *D_Gt_*, *D_Cant_*, *D_Tw_*, *D_Iv_*, *D_Ih_*, and *D_GGt_* formed the basis for calculating the five-parameter defectiveness *D_5_* and six-parameter defectiveness *D_6_*, whereas *D_5_* did not include the defectiveness of the track gauge gradient *D_GGt_* when applying Equation (1). Meanwhile, the new defined parameter included the defectiveness of the track gauge gradient *D_GGt_* in Equation (2).

The new defined and implemented six-parameter defectiveness parameter *D_6_* represents a new approach in monitoring the condition of railway track geometry and its suitability for the assumed exploitation conditions. Relative assessments are focused on the defectiveness and indicators of the individual geometric parameters of the railway track. The six-parameter defectiveness parameter *D*_6_ informs one about the real state of the geometric system, thus illustrating how close it is to smoothly running. Therefore, this parameter can indicate the extent to which the running railway rolling stock on a railway track’s geometric state configuration, for a certain speed, does not cause vibrations, especially in regard to those vibrations that adversely affect the passenger.

The group of absolute assessments includes the synthetic indicator of track condition *J*. Indicator *J* is an objective assessment of railway track condition, independent of the speed allowed on the railway line, and is defined by regulation Id-14 (D-75) [42], which complies with the requirements of the Railway Transport Act of 28 March 2003 [1], under the directives of the European Union. Unlike relative assessments, the absolute assessment of a railway track condition does not depend on the speed of trains and does not depend on the permissible deviations of individual geometric parameters of the railway track condition that are applicable to a given railway track’s baseline sections. The *J* indicator is calculated on the basis of standard deviation *σ_i_* of the parameters considered in Equation (4) [42]:

(4) $$J=\frac{\sigma_{Iv}+\sigma_{Ih}+\sigma_{Tw}+0.5\sigma_{Gt}}{3.5}$$

where *σ_Iv_*—standard deviation of the vertical irregularities; *σ_Ih_*—standard deviation of the horizontal irregularities; *σ_Tw_*—standard deviation of the track twist; *σ_Gt_*—standard deviation of the track gauge.

When conducting calculations of the synthetic track condition indicator *J*, according to Equation (4), the standard deviations *σ_i_*, as recommended by Id-14 (D-75) [42], are defined by Equation (5):

(5) $$\sigma_{i}=\sqrt{\frac{1}{n}\overset{n}{\underset{i=1}{\sum }}{\left(X_{i}-x\right)}^{2}}$$

where *n*—the number of recorded signals on the analysed railway track baseline section; *X_i_*—the value of the parameter at point *i*; *x*—average value of the signal.

The values of the *J* indicator do not independently determine the maximum train speeds. They also depend on complying with all the permissible deviations. According to regulation Id-14 (D-75) [42], the maximum speed of the train may be limited by the value of the *J* indicator when it is not limited by any permissible deviation. This indicator has an impact on the restrictions being implemented. Among other things, the absolute assessments are conducted with standard deviations and a synthetic track condition indicator, which is a function of the standard deviations of irregularities. 

Estimates of the railway track condition have applications in performing track condition analyses and assessments. The synthetic *J* indicator and the defectiveness parameters *D_5_* and *D_6_* complement each other, thus constituting their widespread use. This is achieved by providing information about configuration of the geometric state of the railway tracks in the sustainability development of the ETS. As the state-space interface of the railway tracks’ geometry system with the electrified traction system is its geometric configuration, it is important to maintain comfort and smooth operation in correspondence with the ETS. Furthermore, such an approach should be the priority for wheel/rail and pantograph/catenary interactions and for safety.

### 2.2. Electrified Traction Systems 

#### 2.2.1. Catenary

Catenary is an electrical system by which power is provided to traction vehicles (i.e., catenary-supplied electric vehicle, non-autonomous electric vehicle). A catenary consists of cable assemblies with supporting structures for the overhead contact line and return network.

The contact line is a part of the catenary and is installed over the railway track. It is composed of an assembly of wires and cables, or profiles, together with supporting structures. Additionally, it is responsible for both mechanical and electrical interaction with the current collectors (pantograph, pantograph system current collector) of the electric traction vehicles. The running basis is the configuration of the geometric state of railway tracks in the sustainability development of electrified traction systems. The contact line for the direct supply of electricity to a traction vehicle via current collectors consists of a wiring harness with network accessories and support structures [15].

In contrast, the return network (RN) is also part of the catenary. It is part of the electricity supply circuit. This energy is supplied from the traction substation to a rail vehicle. RN contains a circuit segment that is connected on one side to the return pole in the traction substation, and on the other side to the wire in contact with the rail vehicle. The return network consists of the rails of the railway track and their electrical connections that carry the traction current [15]. At the same time, metal above-ground structures, located in the strip of the railway line at a distance of less than 5.0 m from the axis of the outermost electrified railway track, should be connected to the return network [15]. Legal regulation [15] also defines further mandatory requirements, which are required to be met for the railway rails. Accordingly, railway rails that are not welded in the CWR track should be connected with bondings that are attached to the rail by welding, soldering, pressing, bolting, or any other method that is approved by the railway administration.

The catenary may appear as an overhead catenary. Additionally, this type of network is present on all the real objects of the scientific research work that was carried out for this publication, in which the configuration of the geometric state of railway tracks in the sustainability development of electrified traction systems is very important. It is worth noting that the catenary can also exist as a bottom catenary. It is generally made in the form of a third rail called a current rail and is applicable to subway or underground rail systems, e.g., the subway in Berlin (germ. U-Bahn Berlin); or a next high-speed urban railway, e.g., in the S-Bahn in Berlin (germ. S-Bahn Berlin GmbH). The fourth rail can also be used as a return network, e.g., as is the case in the London Underground.

**Figure 3:**
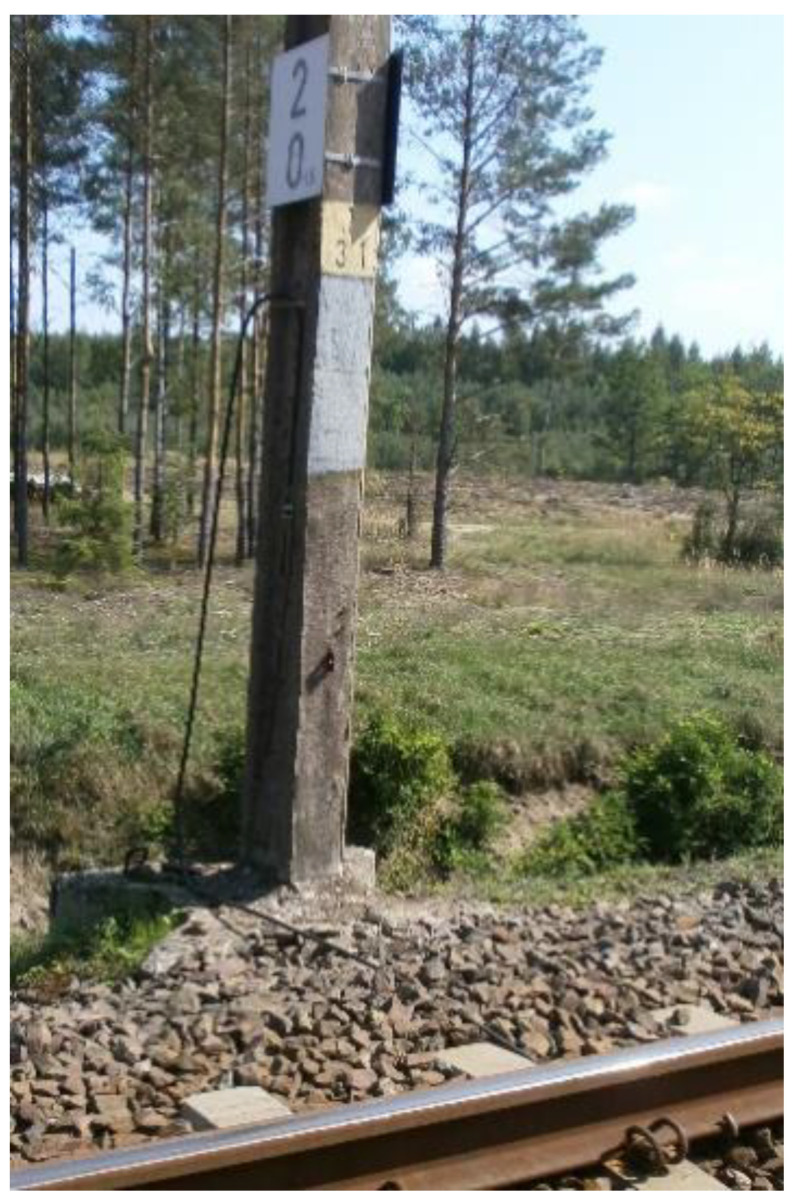
Return network of a DC traction system in object Nos. R_143_1_1_ and R_143_1_2_.

The return network of a DC traction system, operating at all real facilities of the scientific research work carried out in this publication, includes return cables and rails, along with the connections between them (Figure 3). 

#### 2.2.2. Suspension Height of the Contact Line, Contact Line Stagger—The Offset and Stagger of the Contact Line—Zigzagging

**Figure 4:**
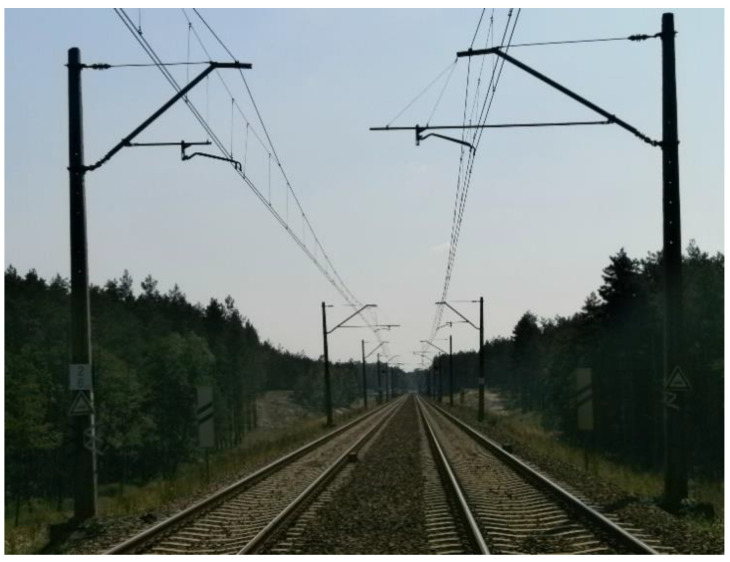
An example of a state-space interface of the railway track geometry system with the electrified traction system, the geometric configuration of object Nos. R_143_1_1_ and R_143_1_2_.

Contact line geometry includes the parameters of the contact line, specifying, in particular, the suspension height of the contact wires and the lateral deviation’s contact line stagger of the contact wire with respect to the track axis (railway track axis, railway track centreline). Additionally, these are correlated with the architecture of the geometric parameters of the railway track (Figure 4).

The suspension height of the contact line is the vertical distance from the contact wire to the plane, connecting the running surfaces of the rails. This was constructed in accordance with the recommendations of the legal regulation Iet-2, measured at the railway track axis [43]. This height on the real objects is included in the scientific research work (interpreted as the contact wires of the catenary of the main railway track and the main additional railway tracks) and should be contained in the range of 4900 ÷ 5600 mm above the plane of the rail heads (crown of the rail). With that being said, the normal (projected) suspension height of the contact wires is 5200 ÷ 5600 mm [43]. The suspension heights of the contact line should not be confused with the structural height of the network, as the structural height of the network is the vertical distance between the suspension line and the contact wire at the point of suspension [43].

**Figure 5:**
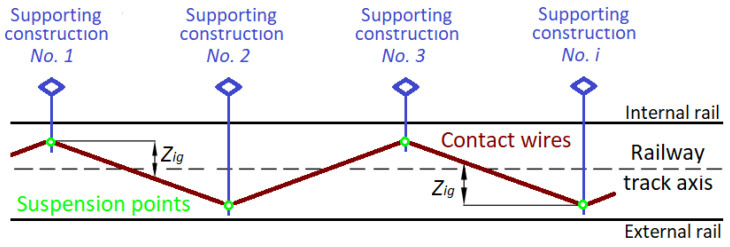
Stagger of the contact line.

The contact line stagger is a structural offset of the contact wire and the contact line from the railway track axis at the point of suspension of the contact line [43] in the horizontal plane. As a result of alternate staggering, the stagger of the contact line (zigzagging, zigzagging of the network) is achieved. A stagger of the contact line gives the contact wires offset *Z_ig_*, both from and to the pole (supporting construction of traction contact line, supporting construction), the points of successive suspensions (Figure 5).

### 2.3. Scientific Research and Industry-Specific Methods

**Figure 6:**
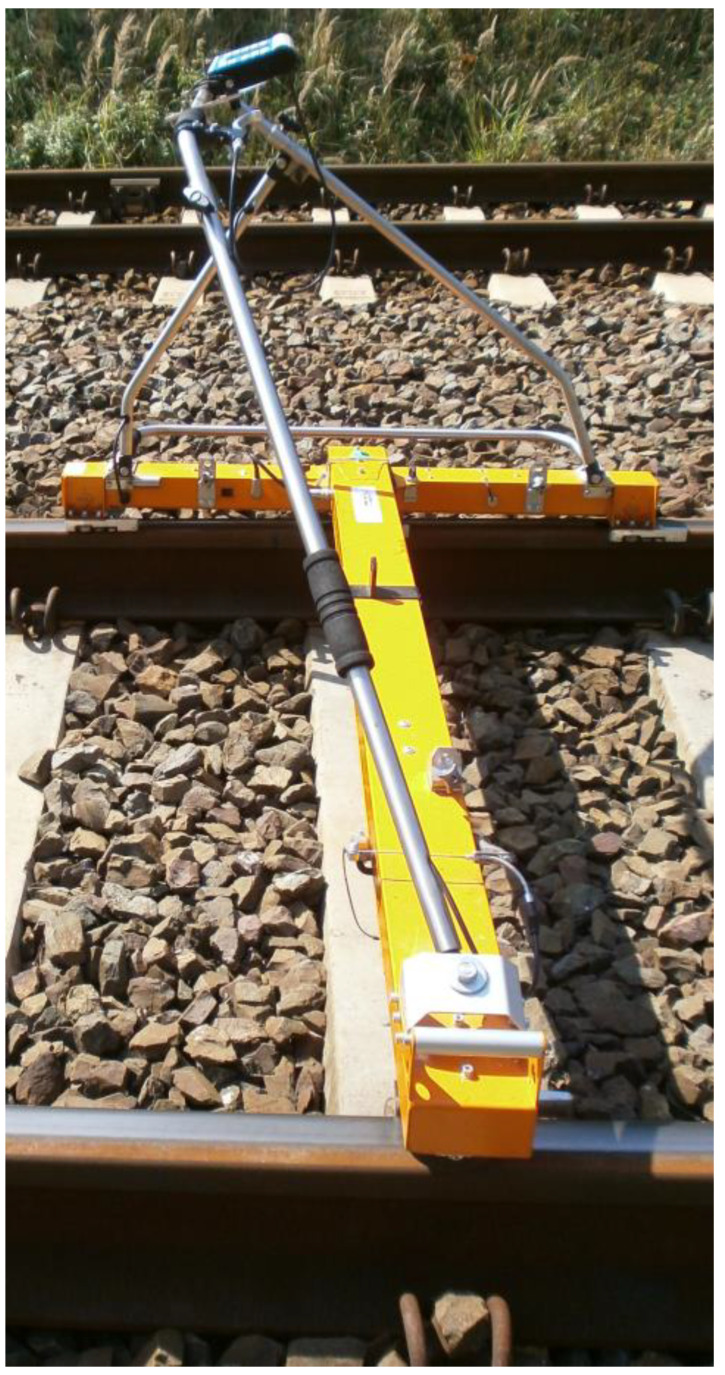
Electronic self-recording track gauge TEC-1435 N2.

The implementation of the scientific research work configuration of the geometric state of railway tracks in the sustainability development of the ETS required the selection of appropriate scientific research and industry-specific methods. In the scientific research and industry-specific process, the choice of methods was one of the most important steps. It is highly beneficial when scientific research work is integrated with methods that are applicable to the industry. This approach determines the meaningfulness and, particularly importantly, the matching of the data obtained; furthermore, the results are also delivered in accordance with reality. The work carried out focused on the geometric state of railway tracks in the sustainability development of the ETS, in which the materials and methods were adapted such that the end result is a valuable and innovative tool. The work carried out included the integration of the following:

- The direct measurements method (DMM). This was conducted in interaction with fixed-point method (FPM), visual method (VSM), and expert methods (EM). When specifically using measuring instruments, the electronic self-recording track gauge TEC (TEC-1435 N2—Figure 6) and trackscan clearance (TSC (Laser-TEC)) should be utilised. These are insulated instruments. When utilising these isolated instruments (this feature is particularly important for the implementation of measurements on electrified railway lines with a return network), then these should be qualified for surveying and diagnostic work.

- The construction of the measuring instruments is three-point. In addition, their measuring elements consist of linear displacement induction sensors (measuring track gauge, horizontal and vertical irregularities) and an electronic level (measuring cant). The measurements made it possible to obtain the values of railway track geometry parameters in the horizontal plane *H*, specifically with track gauge *Gt*, gradient of track gauge *GGt*, and horizontal irregularities *Ih*. Regarding the parameters in the vertical plane *V*, there are cant *Cant* (position of the track in the cross-section), twist *Tw*, and vertical irregularities *Iv*. The modular construction of the measuring instruments was integrated with the laser head that was installed on the electronic self-recording track gauge, which made it possible to measure the suspension height of the contact line and the stagger of the contact line. The survey work also included the monitoring of such elements as the cracks and local depressions of the rolling surface (squat), lack of bolts, condition of the prism, condition of the fastenings, etc. These were based on the method of visual inspection of the permanent way, especially in respect of the visual inspection of visible permanent-way elements. In addition, rail displacement measurements, using the fixed-point method, were taken from the CWR track because it is a location that is susceptible to the creeping of rails [13,14,44]. The purpose of this method was to determine the value of the creeping of the rails with respect to fixed-points. Measurements were carried out on all the railway tracks of the research objects. The interactions of the DMM and FPM with the VSM and EM are based on the purposeful selection of people involved in scientific research work and industry-specific processes, along with the recommendation of future options in terms of their conduct. The applications of the expert methods are also referred to by Dedík et al. in [45], whereby they deal with the research assessment of the perspective ratios in rail crossings as an important evaluation factor of rail crossings.
- Brainstorming method (BM). This approach is known as a creative method that is utilised to solve various problems via using the generation of progressive ideas and thoughts. Furthermore, the result should be an original and unique solution to a specific problem [45]. Antoszkiewicz in [46] emphasises that the brainstorming method is a method for seeking ideas in many sectors of business and administration and in any manifestation of personal life.
- Mind mapping method (MMM). This method develops the BM, through which the logic of the researched problem, context, and priorities are developed [45].
- System approach method (SAM). This is a method that emphasises the overall picture and the interrelationships and connections between the individual components of the whole. It can be called the science of management, of decision-making, or the science of systems thinking [45]. The uniformity and desirability of the system, which is made up of interrelated parts, is emphasised.
- Heuristic method (HM). This is an important tool in various fields of human activity. Due to its high efficiency and effectiveness in arriving at a result, it constitutes a set of methodological recommendations for the search for ideas and the development of new solutions and is aimed at developing innovations [47]. The method offers and discovers new ways of solving problems and inventing certain new contexts. It is a scientific activity based on a ‘discovery’ procedure, which usually starts with a general proposal or some rough estimate, which is then gradually refined. This method represents an intersection between empirical and exact methods [45,48].
- Failure mode and effect analysis (FMEA) method. This approach belongs to the basic group of analytical methods that are used in the quality management process, management reliability, and security [45]. Ben-Daya in [49] emphasises that the FMEA is a systematic analysis of the potential failure modes that are aimed at preventing failures. It is intended to be a preventive action process that is carried out before implementing something new or for changes in products or processes [49].
- System failure mode effects analysis (SFMEA) method. Piechowski et al. in [50] noted that the FMEA method has already been modified and refined several times. These modifications depended on the following: the domain in which it was used, e.g., the EFMEA environmental analysis (environmental FMEA); the business areas of the company, e.g., an analysis of the SFMEA system (system FMEA); and the function performed, as in the FMECA (failure mode and criticality analysis). At the same time, Dedík et al. in [45] state that the SFMEA helps with analysing systems and subsystems at an early (conceptual) stage. In addition, it focuses on interactions between systems and system elements. These issues are also addressed by Rausand and Hoylan in [51], when referring, in particular, to system reliability theory.
- Based on a case study (CSM). Crowe et al. in [52] state that the case study approach allows in-depth, multi-faceted explorations of complex issues in their real-life settings. This is particularly useful to employ when there is a need to obtain an in-depth appreciation of an issue, event, or phenomenon of interest in its natural real-life context.

## 3. Results and Discussion

### 3.1. Space Interface Statistics Railway Track Geometry and the ETS

**Figure 7:**
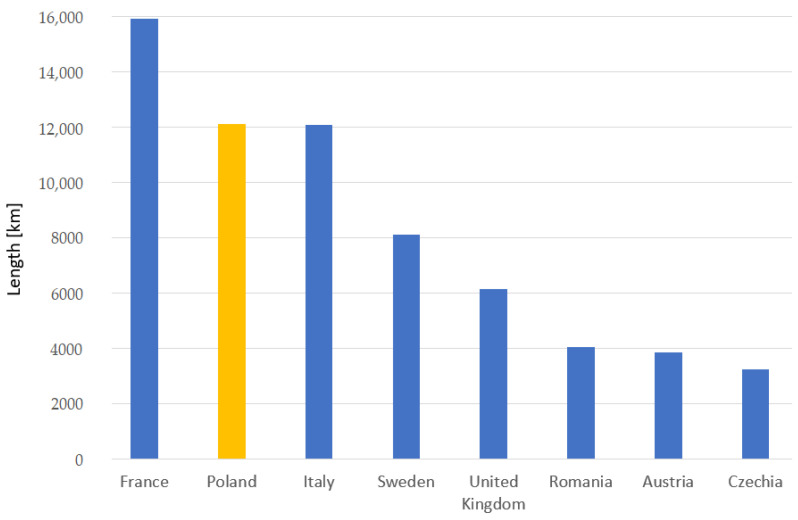
Correlation number of the electrified lines of rail transport, relative to the standard railway track gauge parameter in 2020. Yellow colour—Poland; blue colour—other European countries.

Transportation infrastructure (transport infrastructure) is essential for the development of the economy and society. Transportation statistics are evolving significantly while responding to policy needs, while maintaining coverage and the quality of existing datasets [53]. Rail transport infrastructure is an important sector economy. At the same time, it also represents a pivotal role in mobility and sustainability development. The scientific research work in conjunction with the practice of this publication was carried out in the territory of Poland. With reference to the location of the scientific research objects, Figure 7 shows a comparison with other countries of the number of electrified lines of the rail transport that is relative to the standard railway track gauge parameter in 2020 [53]. This statistic is reflected in the subject of ongoing scientific research work for the configuration of the geometric state of railway tracks in the sustainability development of the ETS.

**Figure 8:**
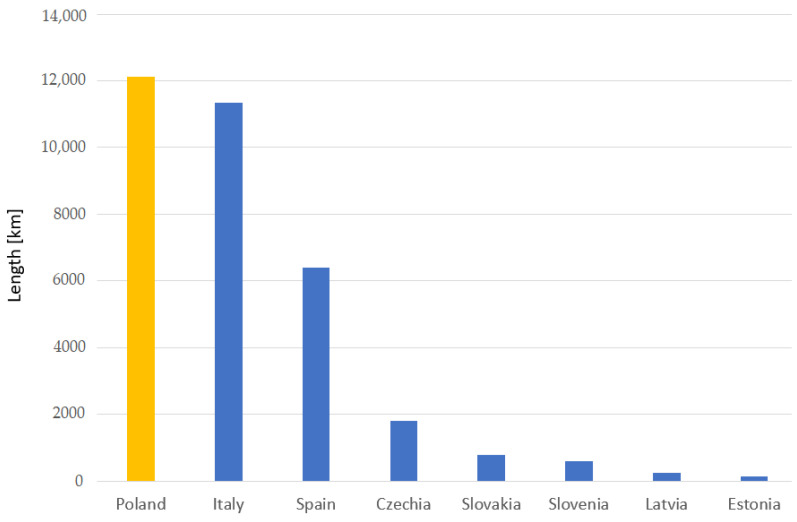
Correlation number of the electrified lines of rail transport relative to the type of voltage, i.e., direct current, 3000 V in 2020. Yellow colour—Poland; blue colour—other European countries.

**Figure 9:**
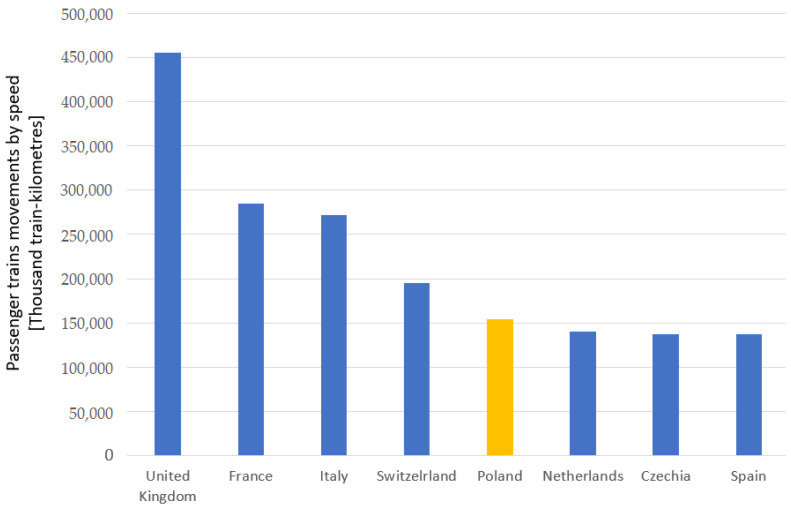
Correlation of the overall passenger train traffic versus speed in 2020. Yellow colour—Poland; blue colour—other European countries.

At the same time, when revealing what the state of dependence of the number of electrified lines of rail transport (rail transportation) is in relation to the type of voltage, a direct current of 3000 V in 2020 is significant especially in Poland as it amounts to 12,111 km, whereas in Italy it is 11,332 km (Figure 8) [53]. Thus, the relationship of overall passenger train traffic in relation to speed (Figure 9), when compared to other countries, presents Poland with 153,906 (thousand train kilometres) [53]. 

### 3.2. Case Study and Field Measurements

#### 3.2.1. Characteristics of the Research Objects

Measurement work was carried out on three real objects (object Nos. R_143_, R_144,_ R_161_), which were represented by the three railways being studied in exploitation. This covered single- and double-track rail routes (railway tracks in open line), which are located in Poland. The real objects were a total of five research objects, specifically railway tracks (object Nos. R_143_1_1_ and R_143_1_2_, R_143_1_2_, R_144_1_, R_161_1_, R_161_2_). These objects are characterised by different geometric features in plan and profile, as well as operational elements of transport engineering (transportation engineering) (Table 1) [54]. The scientific research work was carried out from 2019, and four time horizons were achieved (*M_1_* ÷ *M_4_*; *M_1_* = 2019, *M_2_* = 2020, *M_3_* = 2021, *M_4_* = 2022):

- Object Nos. R_143_1_1_ and R_143_1_2_—Railway track No. 1: *M_1_* ÷ *M_4_*;
- Object No. R_143_1_2_—Railway track No. 2: *M_1_* ÷ *M_4_*;
- Object No. R_144_1_—Railway track No. 1: *M_1_* ÷ *M_4_*.

From 2021, two time horizons were achieved (*M_3_* ÷ *M_4_*; *M_3_* = 2021, *M_4_* = 2022):

- Object No. R_161_1_—Railway track No. 1: *M_3_* ÷ *M_4_*;
- Object No. R_161_2_—Railway track No. 2: *M_3_* ÷ *M_4_*.

#### 3.2.2. Results of the Study

As a result of the scientific research work configuration of the geometric state of railway tracks, specifically in regard to the sustainability development of the ETS, the estimates of the railway track condition are detailed in the materials below:

- Absolute *J* (Appendix A);
- Relative *D_5_* (Appendix B);
- Relative *D_6_* (Appendix C).

These appendices detail all five research objects, specifically the railway tracks (object Nos. R_143_1_1_ and R_143_1_2_, R_143_1_2_, R_144_1_, R_161_1_, R_161_2_). Depending on the geometric parameter to be monitored, the basic measurement cycle was cycled every 0.5 m. The analysis and assessment of the track’s condition was carried out for 100 m long base sections for each of the research objects, i.e., the railway tracks. The data obtained were used for the relative and absolute assessments.

**Figure 10:**
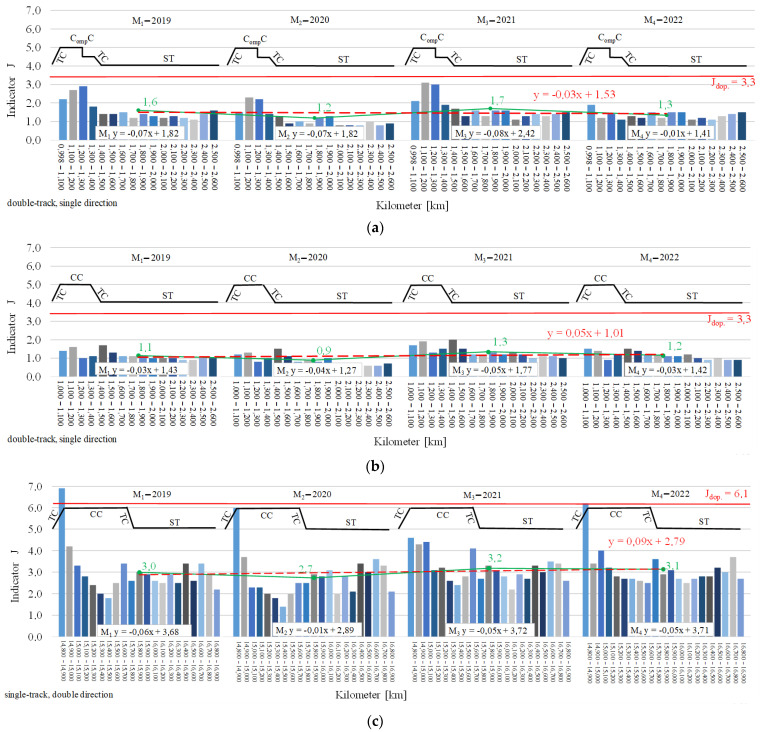

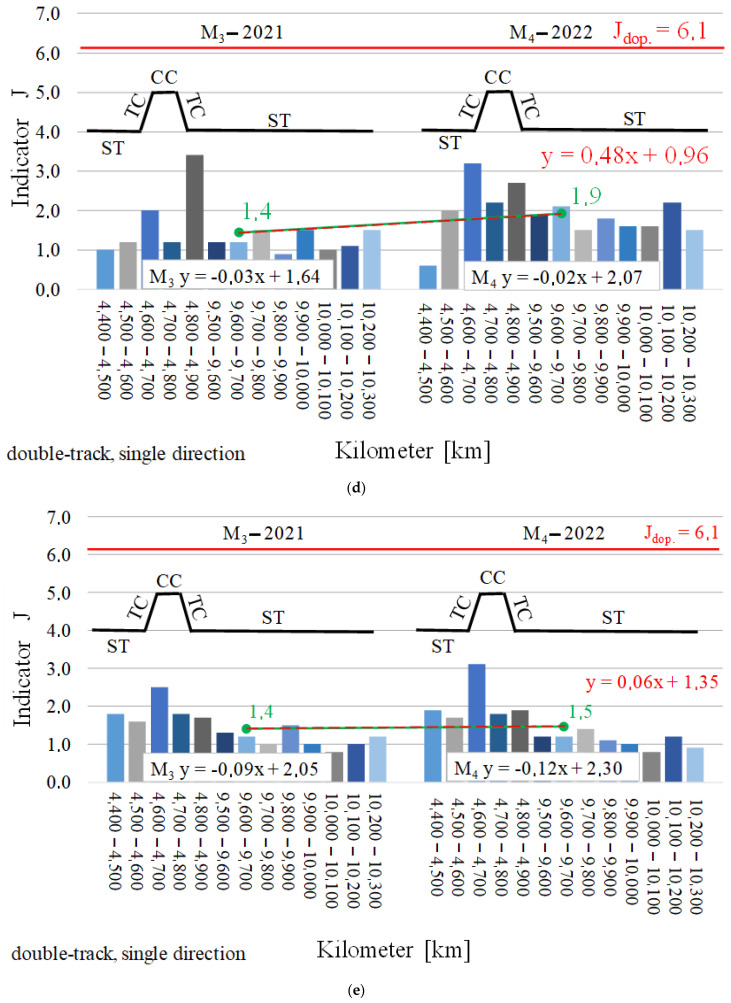
Confrontation of the absolute assessment (absolute estimates) *J* in time horizons *M_1_*_÷_ *M_4_*: (**a**) Object Nos. R_143_1_1_ and R_143_1_2_; (**b**) object No. R_143_2_; (**c**) object No. R_144_1_; (**d**) object No. R_161_1_; and (**e**) object No. R_161_2_. Abbreviations: transition curve—*TC*; compound —*C_omp_ C*; circular —*CC*; straight segment; straight linear segment—*ST*; and time horizon—*M_i_*.

A graphical interpretation of the confrontation of the absolute assessment of *J*, in the time horizons of *M_1_* ÷ *M_4_*, is provided in Figure 10. The individuated bars show the absolute assessment values of *J* for the 100 m baseline sections. The line graph—marked in black—provides a link to the site plan, thereby indicating the location of the *J* assessment on curvilinear segments, i.e., the transition curves (*TC*), circular curves (*CC*), compound curves (*C_omp_ C*), or straight segments (straight-lined segments, *ST*). For each time horizon *M_1_* ÷ *M_4_*, the linear correlations are also presented for all objects, *M_1_ y* ÷ *M_4_ y*. The graphs of Figure 10 for the individual objects also include a line graph, whereby the red colour indicates the allowable value of the absolute *J_dop_* assessment (the allowable value of the absolute *J_dop_* estimates). The scientific research work has shown that on all research objects the highest values of the absolute *J* assessment occur on the following: curvilinear segment, i.e., transition curves, circular curves, and compound curves (the maximum values of individual pole peaks). The *ST* straight-lined segments were characterised by lower values, as reflected in the regression equations for the individual measurement periods of *M_1_* ÷ *M_4_* (Figure 10).

In addition, the scientific research work shows that the mean values of the absolute assessment *J*—indicated by the line graph in green—gradually increase or decrease with their values in the subsequent measurement periods of *M_1_* ÷ *M_4_*. The differences indicate the effects of the preventive maintenance and corrective maintenance, thus having a grounding in the quality of the track’s maintenance condition and thereby resulting in an impact on sustainability development (Figure 10a–c). On the other hand, in the graphs contained in Figure 10d,e, the average values of the *J* rating in the successive measurement periods increase. Therefore, the quality of the maintenance condition of the railway track layout configuration decreases. At the same time, in almost all analysed objects, the fitted regression function in the average values of *J* assessment for particular measurement periods is increasing (Figure 10). The exception is the equation determined from the measured data for object Nos. R_143_1_1_ and R_143_1_2_ (Figure 10a), which was characterised by inadequate maintenance during covered by the scientific research work period. The analytical data of the absolute assessment values *J* are included in Appendix A.

**Figure 11:**
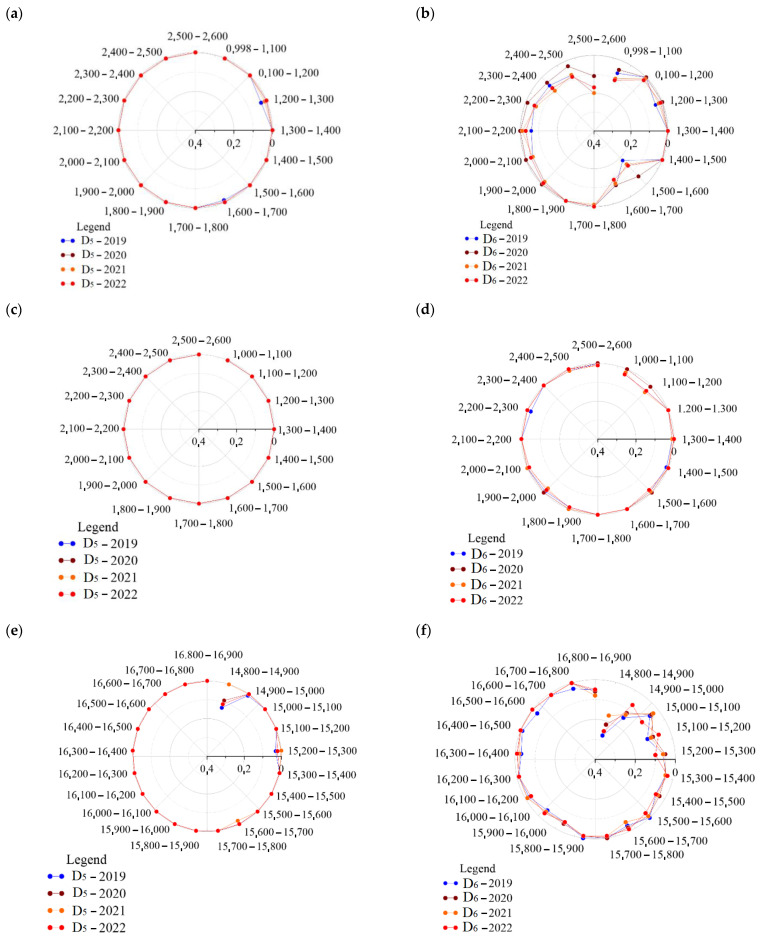

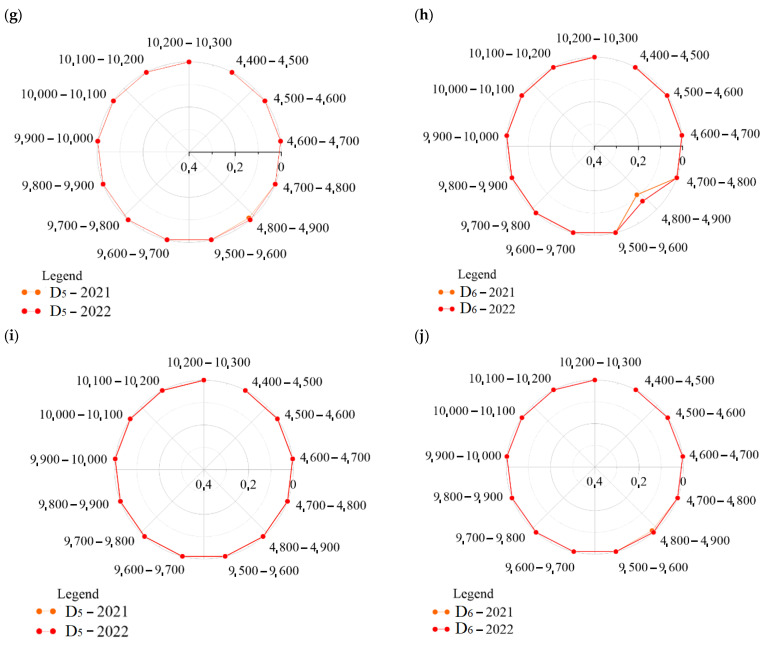
Confrontation of the relative assessment (relative estimates) *D_5_* and *D_6_* parameters in the time horizons of *M_1_*_÷_ *M_4_*: (**a**) *D_5_* object Nos. R_143_1_1_ and R_143_1_2_; (**b**) *D_6_* object Nos. R_143_1_1_ and R_143_1_2_; (**c**) *D_5_* object No. R_143_1_2_; (**d**) *D_6_* object No. R_143_1_2_; (**e**) *D_5_* object No. R_144_1_; (**f**) *D_6_* object No. R_144_1_; (**g**) *D_5_* object No. R_161_1_; (**h**) *D_6_* object No. R_161_1_; (**i**) *D_5_* object No. R_161_2_; and (**j**) *D_6_* object No. R_161_2._

A graphical interpretation of the confrontation of the relative assessment of *D_5_* and *D_6_*, at time horizons *M_1_* ÷ *M_4_*, was provided in Figure 11. The track defectiveness, as a relative measure of the railway track, was dependent on the maximum speed of the trains (Table 1). In addition, it expresses the ratio of the length of railway track where the permissible deviations are exceeded in regard to the total length considered. The scientific research work carried out confirms, in terms of the analysis and assessment of the *D_5_* and *D_6_* values, that the new defined and implemented six-parameter *D_6_* defectiveness parameter for the railway track condition estimates has a significantly higher sensitivity than *D_5_*.

The graphical representation of the correlation of the dependence of this phenomenon, followed by its variability, was confirmed on the basis of the acquired data for all the research objects in the time horizons of *M_1_* ÷ *M_4_*. The existing parameter *D_5_* is lenient, while not reflecting directly the real state of track defectiveness (Figure 11a,c,e,g,i). On the other hand, the numerous instances found regarding the relative measure of track condition *D_6_* provides reliable information about the existing railway track defectiveness (Figure 11b,d,f,h,j). These results suggest that the *D_6_* relative assessment allows for a more in-depth analysis and assessment of the railway track condition, thus allowing for a correctly addressed identification of the defectiveness railway track baseline sections.

In addition, the values of parameter *D_6_* are significantly higher on curvilinear segments, with the values of *D_5_* mostly oscillating around 0 on the same segments. Appendix B includes estimates of the railway track conditions, specifically in relation to *D_5_*, and Appendix C includes estimates of railway track conditions relative to *D_6_*.

The contact wires of the catenary of the railway tracks on the open line, specifically in respect of the research objects that are suspended between 4900 ÷ 5600 mm above the plane of the rail heads, were investigated. In the scientific research work that was carried out on all the research objects, the train speeds were between 70 ÷ 120 km/h (Table 1). This height was in accordance with the suspension height of the contact wires that resulted from the technical documentation, with a tolerance of the suspension height of the contact wires for railway tracks with the following running speeds [43]:

- 100 ≤ *V_i_* ≤ 160 km/h is 0 ÷ 50 mm;
- *V_i_* < 100 km/h is 0 ÷ 100 mm.

The contact line stagger, i.e., the contact wire gauge for the scientific research object, is within the tolerances. This was, according to [43], for railway lines with a running speed of *V_i_* ≤ 160 km/h, as follows:

- 300 ± 80 mm in the direction from or to the pole in a straight line;
- 400 mm on the curve with a tolerance of +20 mm towards the outside of the curve and −60 mm towards the inside of the curve, whereby the centre line of the contact wires must be tangential to the axis of the railway track at the centre of the through intermediate suspension bay.

## 4. Discussion

The scientific research work carried out was implemented on the rail transport infrastructure. In addition, the safety developments of civil engineering, surveying/geodesy, and transport were also investigated. At the same time, this study fits into an existing global scientific research gap, including and ensuring the definition and implementation of the six-parameter *D_6_* defectiveness parameter for the purposes of railway track condition estimates. Açikbas and Söylemez in [26] specifically stated that the degree of energy use depends on the following parameters:

- Train operation frequency (headway);
- Train-set auxiliary power consumption rate;
- Nominal braking acceleration rate;
- Braking effort vs. velocity curve of trains.

In addition, the power system configuration, such as the substation locations and distances, catenary system resistance, and nominal power feeding voltage level, also have a considerable effect on the recuperation rate. The findings of the study [26] were complemented by the results of this scientific research work on the realised subject of configuration regarding the geometric state of railway tracks in the sustainability development of the ETS. Thus, the present scientific research work corresponds and correlates with the output of international researchers, filling a hitherto unfilled gap in this scientific research area. At the same time, this study detailed the increase in the interoperability of railway tracks’ geometric state configurations in the sustainability development of the ETS. This was achieved by ensuring that the six-parameter defectiveness *D_6_* was defined and implemented in respect of the railway track condition estimates. The new approach reinforces the improvement of preventive maintenance and the reduction in corrective maintenance. It represents an innovative supplement to the existing direct and next indirect measurement method in the configuration of the geometric condition of railway tracks in the sustainability development of the ETS. Thus, the realised subject is a complementary element of the supporting architecture, which is achieved by supporting the existing condition and having a base at the heart of the entire rail transport system. Bhatti et al. in [55] recognises the requirements of sustainable transport that complies with the United Nations’ Sustainable Development Goals. However, more importantly, it emphasises the requirement for a supporting architecture. This is because it should contribute to optimisation.

In terms of civil engineering, surveying/geodesy, and transport—especially in respect of rail transport and in realising their development—the six-parameter defectiveness *D_6_* has been defined and implemented in regard to the railway track condition estimates. When presenting the reality, it is particularly important, not only during the exploitation process but also in the design or modernisation process, to include DC railways in the assessment [56]. Tian et al. in [56] also note that the modelling and simulation of the electrified transit system is an essential element in the design process of a new railway, or in an existing one being modernised. The problems of evolution were addressed by Judek et al. in [57], whereby they emphasised the requirements of monitoring rail transport infrastructure by using ‘hand-held’ and ‘sensor-based’ technologies for ‘Industry 4.0’ [58]. Additionally, this study was also characterised by the application of information and communication technologies, particularly in the rail industry. The subject of the relevance of evolution is also highlighted by Kostrzewski and Melnik in [59], especially in the interaction of rail vehicle subsystem and track subsystem monitoring. They also see the evolution of these approaches, whereby it ‘has followed manual maintenance, through methods connected to the application of sensors, up to the currently discussed methods and techniques focused on the mutual use of automation, data processing, and exchange’. Evolution providing a representation of the reality of the configuration of the geometric state of railway tracks in the sustainability development of the ETS is due to its detail being fully representative of the relative estimates of railway track conditions.

The state-space interface of the railway track geometry system, with the electrified traction system, is found in its geometrical configuration, which not only has a significant role to play during exploitation but also in terms of processes such as the regulation of railway track axes. This is achieved as a result of the requirement to coordinate activities by having a common denominator. The new approach, particularly through the implementation of parameter *D_6_*, reinforces the improvement of preventive maintenance and the reduction in corrective maintenance, thus complementing the existing surveying and diagnostic technologies in the correct configuration of the geometrical state of the railway tracks in the sustainability development of the ETS.

The results of the work carried out have confirmed their relevance. The new approach, representing the definition, equation, and implementation of the six-parameter defectiveness parameter *D_6_* for the estimates of railway track conditions, fits in with the innovation, as well as in the implementation of a new and significantly improved parameter—especially in regard to processing the estimates. The new approach is representative of the progress of knowledge, especially regarding the real configuration of the geometric state of railway tracks in the sustainability development of ETSs; in addition, it provides constructive information. It also increases the interoperability configuration of the geometric state of railway tracks in the sustainability development of ETSs. The implementation of the new approach is an innovative contribution to the development of the engineering and technical field, particularly in the discipline of civil engineering, surveying/geodesy, and transport, thus creating new opportunities in contributing to sustainability development. Furthermore, this study represents smarter and more efficient applications in regard to the integration configuration of the geometric state of railway tracks in the sustainability development of ETSs, thereby leading to progress and the reinforcement of the dynamics of their correlation.

## 5. Conclusions

In this scientific research work, the configuration of the geometric state of railway tracks in the sustainability development of the ETS is presented. This subject is particularly important in terms of the correlation of driving comfort, smooth operations, and in the interactions with the ETS. As a result of the definition and implementation in regard to the new parameter of the so-called six-parameter defectiveness *D_6_*, specifically as a parameter for railway track condition estimates, the new approach reinforces the improvement of preventive maintenance and reductions in corrective maintenance. In addition, this study shows innovation with respect to the existing direct measurement method. This will, in turn, result in indirect measurement methods on railway track geometric state configurations in terms of contributing towards the sustainability development of the ETS. All the configurations used in this study are deterministic; thus, the results of the scientific research work are robust. The new approach used in the analysis and assessment of the conditions of railway track geometry configurations, especially in regard to the group of relative assessments, also stands out in terms of the explainability of the existing and real state of the space interface. This reliability is particularly important in the context of railway track geometry and their axis (railway track centreline) in interaction with the suspension height of the contact line, contact line stagger, and stagger of contact lines, i.e., zigzagging. The developed subject matter is well-established in increasing the interoperability of railway tracks geometric state configurations in ETS sustainability development. The results of the work confirmed their validity.

Ensuring the safe movement of traction vehicles on the designated railway route is the responsibility of the railway tracks, whose geometry is compatible with the catenary (primarily in respect of the contact wires). In turn, the contact rails, as part of the railway track, are also a component of the return network, thus also being a part of the catenary. Therefore, increasing the state interoperability of the geometric configuration of the railway tracks in the sustainability development of electrified traction systems contributes to a more efficient maintenance management.

This scientific research work also confirmed that the interactions of the applied scientific research and industry-specific methods allow for a multifaceted analysis and assessment of the assumed issues in a natural and real context.

In future, the results obtained can be used in interaction with a road map regarding the rail transport infrastructures of various determinants. For example, by applying different information to the layers, such as the state-space interface of the railway tracks geometry system with the ETS and its geometric configuration, then there are changes in relation to the follow-up action and in respect of the infrastructure gauge.

## Figures and Tables

**Table 1:** Characteristics of the real and research objects.

Characteristics of a Curvilinear and Straight Segment	Kilometre (km)	Geometry of the Curvilinear Segment	Characteristics of the Research Object			Time Horizons for Measurement
			Speed (km/h)	Category of Railway Line	Type of Railway Line/Movement	
Research object: Object Nos. R_143_1_1_ and R_143_1_2_						
Curvilinear segment:			*V_max_* = 120 *V_ft_* = 100	Prime	Double track, single direction	*M_1_* ÷ *M_4_*
Transition curve Compound curve Transition curve	1 + 006.22 ÷ 1 + 036.22 1 + 036.22 ÷ 1 + 357.02 1 + 357.02 ÷ 1 + 417.02	*L_1_* = 30.00 m *D_1_* = 282.80 m *R_1_* = 1720.00 m *D_2_* = 38.00 m *R_2_* = 2050.00 m *L_2_* = 60.00 m *C_ant t._* = 30 mm				
Straight segment:						
Straight	1 + 417.02 ÷ 2 + 517.02	*D_3_* = 1100.00 m				
Research object: Object No. R_143_2_						
Curvilinear segment:			*V_max_* = 120 *V_ft_* = 100	Prime	Double track, single direction	*M_1_* ÷ *M_4_*
Transition curve Circular curve Transition curve	0 + 986.04 ÷ 1 + 056.04 1 + 056.04 ÷ 1 + 304.44 1 + 304.44 ÷ 1 + 454.44	*L_1_* = 70.00 m *D_1_* = 248.40 m *R_1_* = 1740.00 m *L_2_* = 150.00 m *C_ant t._* = 30 mm				
Straight segment:						
Straight	1 + 454.44 ÷ 2 + 554.44	*D_2_* = 1100.00 m				
Research object: Object No. R_144_1_						
Curvilinear segment:			*V_max_* = 70 *V_ft_* = 70	Prime	Single track, double direction	*M_1_* ÷ *M_4_*
Transition curve Circular curve Transition curve	14 + 860.00 ÷ 14 + 980.00 14 + 980.00 ÷ 15 + 640.00 15 + 640.00 ÷ 15 + 760.00	*L_1_* = 120.00 m *D_1_* = 660.00 m *R_1_* = 1090.00 m *L_2_* = 120.00 m *C_ant t._* = 40 mm				
Straight segment:						
Straight	15 + 760.00 ÷ 16 + 860.00	*D_2_* = 1100.00 m				
Research object: Object No. R_161_1_						
Straight segment:			*V_max_* = 70 *V_ft_* = 70	Prime	Double track, single direction	*M_3_* ÷ *M_4_*
Straight	4 + 411.24 ÷ 4 + 611.24	*D_1_* = 200.00 m				
Curvilinear segment:						
Transition curve Circular curve Transition curve	4 + 611.24 ÷ 4 + 651.24 4 + 651.24 ÷ 4 + 707.02 4 + 707.02 ÷ 4 + 747.02	*L_1_* = 40.00 m *D_1_* = 55.78 m *R_1_* = 3300.00 m *L_2_* = 40.00 m *C_ant t._* = 20 mm				
Straight segment:						
Straight Straight	4 + 747.02 ÷ 4 + 847.02 9 + 500.00 ÷ 10 + 300.00	*D_2_* = 100.00 m *D_3_* = 800.00 m				
Research object: Object No. R_161_2_						
Straight segment:			*V_max_* = 70 *V_ft_* = 70	Prime	Double track, single direction	*M_3_* ÷ *M_4_*
Straight	4 + 408.69 ÷ 4 + 608.69	*D_1_* = 200.00 m				
Curvilinear segment:						
Transition curve Circular curve Transition curve	4 + 608.69 ÷ 4 + 648.69 4 + 648.69 ÷ 4 + 706.38 4 + 706.38 ÷ 4 + 746.38	*L_1_* = 40.00 m *D_1_* = 57.69 m *R_1_* = 3300.00 m *L_2_* = 40.00 m *C_ant t._* = 20 mm				
Straight segment:						
Straight Straight	4 + 746.38 ÷ 4 + 846.38 9 + 500.00 ÷ 10 + 300.00	*D_2_* = 100.00 m *D_3_* = 800.00 m				

Abbreviations: *L_i_*—length of the transition curve; *D_ii_*—straight or curve length; *R_i_*—radius; *V_max_*—speed of passenger trains; *V_ft_*—speed of freight trains; *C_ant t._*—theoretical cant value; *M_i_*—time horizon.

## Data Availability

Not applicable.

## Appendix A. Estimates of Railway Track Condition—Absolute *J*

**Table A1:** Estimates of the railway track condition—absolute *J*, object Nos. R_143_1_1_ and R_143_1_2._

Railway Track Baseline Sections	2019	2020	2021	2022
0.998–1.100	2.2	1.5	2.1	1.9
1.100–1.200	2.7	2.3	3.1	1.2
1.200–1.300	2.9	2.2	3.0	1.4
1.300–1.400	1.8	1.4	1.9	1.1
1.400–1.500	1.4	1.3	1.7	1.3
1.500–1.600	1.4	0.9	1.3	1.2
1.600–1.700	1.5	1.0	1.6	1.5
1.700–1.800	1.2	0.9	1.3	1.2
1.800–1.900	1.4	1.2	1.6	1.5
1.900–2.000	1.3	1.3	1.6	1.5
2.000–2.100	1.2	0.8	1.1	1.1
2.100–2.200	1.3	0.8	1.3	1.2
2.200–2.300	1.2	0.8	1.4	1.1
2.300–2.400	1.1	1.0	1.3	1.3
2.400–2.500	1.4	0.8	1.4	1.4
2.500–2.600	1.6	0.9	1.5	1.5

**Table A2:** Estimates of the railway track condition—absolute *J*, object No. R_143_1_2._

Railway Track Baseline Sections	2019	2020	2021	2022
1.000–1.100	1.4	1.2	1.7	1.5
1.100–1.200	1.6	1.3	1.9	1.4
1.200–1.300	1.0	0.8	1.3	0.9
1.300–1.400	1.1	1.0	1.5	1.2
1.400–1.500	1.7	1.5	2.0	1.5
1.500–1.600	1.3	1.1	1.5	1.4
1.600–1.700	1.1	0.8	1.2	1.2
1.700–1.800	1.1	0.9	1.2	1.2
1.800–1.900	1.1	0.8	1.3	1.1
1.900–2.000	1.0	1.0	1.2	1.1
2.000–2.100	1.0	0.7	1.3	1.2
2.100–2.200	1.0	0.6	1.2	1.0
2.200–2.300	0.9	0.6	1.0	0.9
2.300–2.400	0.9	0.6	1.1	1.0
2.400–2.500	1.0	0.6	1.1	0.9
2.500–2.600	1.0	0.7	1.0	0.9

**Table A3:** Estimates of the railway track condition—absolute *J*, object No. R_144_1._

Railway Track Baseline Sections	2019	2020	2021	2022
14.800–14.900	6.9	6.0	4.6	6.2
14.900–15.000	4.2	3.7	4.3	3.4
15.000–15.100	3.3	2.3	4.4	4.0
15.100–15.200	2.8	2.3	3.1	3.2
15.200–15.300	2.4	2.0	3.2	2.8
15.300–15.400	2.0	1.8	2.6	2.7
15.400–15.500	1.8	1.4	2.4	2.7
15.500–15.600	2.5	2.0	2.8	2.6
15.600–15.700	3.4	2.5	4.1	2.5
15.700–15.800	2.6	2.5	2.7	3.6
15.800–15.900	3.0	2.9	3.3	2.9
15.900–16.000	2.9	2.8	3.1	3.1
16.000–16.100	2.6	3.1	2.8	2.7
16.100–16.200	2.5	2.0	2.2	2.5
16.200–16.300	2.9	2.8	2.9	2.7
16.300–16.400	2.5	2.1	2.7	2.8
16.400–16.500	3.4	3.4	3.3	2.8
16.500–16.600	2.6	3.0	3.0	3.2
16.600–16.700	3.4	3.6	3.5	3.0
16.700–16.800	2.9	3.3	3.4	3.7
16.800–16.900	2.2	2.1	2.6	2.7

**Table A4:** Estimates of the railway track condition—absolute *J*, object No. R_161_1._

Railway Track Baseline Sections	2019	2020	2021	2022
4.400–4.500	-	-	1.0	0.6
4.500–4.600	-	-	1.2	2.0
4.600–4.700	-	-	2.0	3.2
4.700–4.800	-	-	1.2	2.2
4.800–4.900	-	-	3.4	2.7
9.500–9.600	-	-	1.2	1.9
9.600–9.700	-	-	1.2	2.1
9.700–9.800	-	-	1.5	1.5
9.800–9.900	-	-	0.9	1.8
9.900–10.000	-	-	1.5	1.6
10.000–10.100	-	-	1.0	1.6
10.100–10.200	-	-	1.1	2.2
10.200–10.300	-	-	1.5	1.5

**Table A5:** Estimates of the railway track condition—absolute *J*, object No. R_161_2._

Railway Track Baseline Sections	2019	2020	2021	2022
4.400–4.500	-	-	1.8	1.9
4.500–4.600	-	-	1.6	1.7
4.600–4.700	-	-	2.5	3.1
4.700–4.800	-	-	1.8	1.8
4.800–4.900	-	-	1.7	1.9
9.500–9.600	-	-	1.3	1.2
9.600–9.700	-	-	1.2	1.2
9.700–9.800	-	-	1.0	1.4
9.800–9.900	-	-	1.5	1.1
9.900–10.000	-	-	1.0	1.0
10.000–10.100	-	-	0.8	0.8
10.100–10.200	-	-	1.0	1.2
10.200–10.300	-	-	1.2	0.9

## Appendix B. Estimates of the Railway Track Condition—Relative *D_5_*

**Table A6:** Estimates of the railway track condition—relative *D_5_*, object Nos. R_143_1_1_ and R_143_1_2._

Railway Track Baseline Sections	2019	2020	2021	2022
0.998–1.100	0.00	0.00	0.00	0.00
1.100–1.200	0.00	0.00	0.00	0.00
1.200–1.300	0.03	0.00	0.01	0.00
1.300–1.400	0.00	0.00	0.00	0.00
1.400–1.500	0.00	0.00	0.00	0.00
1.500–1.600	0.00	0.00	0.00	0.00
1.600–1.700	0.01	0.00	0.00	0.00
1.700–1.800	0.00	0.00	0.00	0.00
1.800–1.900	0.00	0.00	0.00	0.00
1.900–2.000	0.00	0.00	0.00	0.00
2.000–2.100	0.00	0.00	0.00	0.00
2.100–2.200	0.00	0.00	0.00	0.00
2.200–2.300	0.00	0.00	0.00	0.00
2.300–2.400	0.00	0.00	0.00	0.00
2.400–2.500	0.00	0.00	0.00	0.00
2.500–2.600	0.00	0.00	0.00	0.00

**Table A7:** Estimates of the railway track condition—relative *D_5_* object No. R_143_1_2._

Railway Track Baseline Sections	2019	2020	2021	2022
1.000–1.100	0.00	0.00	0.00	0.00
1.100–1.200	0.00	0.00	0.00	0.00
1.200–1.300	0.00	0.00	0.00	0.00
1.300–1.400	0.00	0.00	0.00	0.00
1.400–1.500	0.00	0.00	0.00	0.00
1.500–1.600	0.00	0.00	0.00	0.00
1.600–1.700	0.00	0.00	0.00	0.00
1.700–1.800	0.00	0.00	0.00	0.00
1.800–1.900	0.00	0.00	0.00	0.00
1.900–2.000	0.00	0.00	0.00	0.00
2.000–2.100	0.00	0.00	0.00	0.00
2.100–2.200	0.00	0.00	0.00	0.00
2.200–2.300	0.00	0.00	0.00	0.00
2.300–2.400	0.00	0.00	0.00	0.00
2.400–2.500	0.00	0.00	0.00	0.00
2.500–2.600	0.00	0.00	0.00	0.00

**Table A8:** Estimates of the railway track condition—relative *D_5_* object No. R_144_1._

Railway Track Baseline Sections	2019	2020	2021	2022
14.800–14.900	0.13	0.09	0.00	0.11
14.900–15.000	0.01	0.00	0.00	0.00
15.000–15.100	0.00	0.00	0.00	0.00
15.100–15.200	0.00	0.00	0.00	0.00
15.200–15.300	0.03	0.00	0.00	0.02
15.300–15.400	0.00	0.00	0.00	0.00
15.400–15.500	0.00	0.00	0.00	0.00
15.500–15.600	0.00	0.00	0.00	0.00
15.600–15.700	0.00	0.00	0.02	0.00
15.700–15.800	0.00	0.00	0.00	0.00
15.800–15.900	0.00	0.00	0.00	0.00
15.900–16.000	0.00	0.00	0.00	0.00
16.000–16.100	0.00	0.00	0.00	0.00
16.100–16.200	0.00	0.00	0.00	0.00
16.200–16.300	0.00	0.00	0.00	0.00
16.300–16.400	0.00	0.00	0.00	0.00
16.400–16.500	0.00	0.00	0.00	0.00
16.500–16.600	0.00	0.00	0.00	0.00
16.600–16.700	0.00	0.00	0.00	0.00
16.700–16.800	0.00	0.00	0.00	0.00
16.800–16.900	0.00	0.00	0.00	0.00

**Table A9:** Estimates of the railway track condition—relative *D_5_* object No. R_161_1._

Railway Track Baseline Sections	2019	2020	2021	2022
4.400–4.500	-	-	0.00	0.00
4.500–4.600	-	-	0.00	0.00
4.600–4.700	-	-	0.00	0.00
4.700–4.800	-	-	0.00	0.00
4.800–4.900	-	-	0.01	0.00
9.500–9.600	-	-	0.00	0.00
9.600–9.700	-	-	0.00	0.00
9.700–9.800	-	-	0.00	0.00
9.800–9.900	-	-	0.00	0.00
9.900–10.000	-	-	0.00	0.00
10.000–10.100	-	-	0.00	0.00
10.100–10.200	-	-	0.00	0.00
10.200–10.300	-	-	0.00	0.00

**Table A10:** Estimates of the railway track condition—relative *D_5_* object No. R_161_2._

Railway Track Baseline Sections	2019	2020	2021	2022
4.400–4.500	-	-	0.00	0.00
4.500–4.600	-	-	0.00	0.00
4.600–4.700	-	-	0.00	0.00
4.700–4.800	-	-	0.00	0.00
4.800–4.900	-	-	0.00	0.00
9.500–9.600	-	-	0.00	0.00
9.600–9.700	-	-	0.00	0.00
9.700–9.800	-	-	0.00	0.00
9.800–9.900	-	-	0.00	0.00
9.900–10.000	-	-	0.00	0.00
10.000–10.100	-	-	0.00	0.00
10.100–10.200	-	-	0.00	0.00
10.200–10.300	-	-	0.00	0.00

## Appendix C. Estimates of the Railway Track Condition—Relative *D_6_*

**Table A11:** Estimates of the railway track condition—relative *D_6_*, object Nos. R_143_1_1_ and R_143_1_2._

Railway Track Baseline Sections	2019	2020	2021	2022
0.998–1.100	0.07	0.05	0.10	0.11
1.100–1.200	0.00	0.00	0.01	0.02
1.200–1.300	0.04	0.00	0.02	0.01
1.300–1.400	0.00	0.00	0.00	0.00
1.400–1.500	0.00	0.00	0.00	0.00
1.500–1.600	0.18	0.06	0.15	0.14
1.600–1.700	0.09	0.09	0.10	0.12
1.700–1.800	0.00	0.00	0.01	0.00
1.800–1.900	0.00	0.00	0.00	0.00
1.900–2.000	0.02	0.00	0.02	0.01
2.000–2.100	0.04	0.00	0.04	0.03
2.100–2.200	0.06	0.00	0.01	0.03
2.200–2.300	0.05	0.01	0.06	0.05
2.300–2.400	0.06	0.04	0.10	0.08
2.400–2.500	0.08	0.03	0.08	0.09
2.500–2.600	0.20	0.11	0.20	0.17

**Table A12:** Estimates of the railway track condition—relative *D_6_*, object No. R_143_1_2._

Railway Track Baseline Sections	2019	2020	2021	2022
1.000–1.100	0.03	0.00	0.02	0.03
1.100–1.200	0.04	0.01	0.05	0.04
1.200–1.300	0.00	0.00	0.00	0.00
1.300–1.400	0.01	0.00	0.01	0.00
1.400–1.500	0.01	0.00	0.00	0.00
1.500–1.600	0.01	0.00	0.01	0.02
1.600–1.700	0.00	0.00	0.00	0.00
1.700–1.800	0.00	0.00	0.00	0.00
1.800–1.900	0.01	0.00	0.00	0.01
1.900–2.000	0.01	0.00	0.03	0.02
2.000–2.100	0.00	0.00	0.00	0.01
2.100–2.200	0.00	0.00	0.00	0.00
2.200–2.300	0.02	0.00	0.00	0.00
2.300–2.400	0.00	0.00	0.00	0.00
2.400–2.500	0.01	0.00	0.01	0.00
2.500–2.600	0.00	0.00	0.01	0.01

**Table A13:** Estimates of the railway track condition—relative *D_6_* object No. R_144_1._

Railway Track Baseline Sections	2019	2020	2021	2022
14.800–14.900	0.27	0.22	0.17	0.25
14.900–15.000	0.15	0.12	0.13	0.07
15.000–15.100	0.05	0.04	0.03	0.10
15.100–15.200	0.12	0.09	0.10	0.06
15.200–15.300	0.06	0.05	0.06	0.10
15.300–15.400	0.04	0.04	0.04	0.03
15.400–15.500	0.05	0.03	0.04	0.05
15.500–15.600	0.00	0.01	0.01	0.03
15.600–15.700	0.03	0.02	0.05	0.01
15.700–15.800	0.00	0.00	0.01	0.01
15.800–15.900	0.00	0.01	0.01	0.01
15.900–16.000	0.05	0.04	0.05	0.05
16.000–16.100	0.05	0.04	0.04	0.03
16.100–16.200	0.02	0.01	0.01	0.03
16.200–16.300	0.01	0.01	0.01	0.01
16.300–16.400	0.03	0.01	0.02	0.01
16.400–16.500	0.01	0.00	0.00	0.00
16.500–16.600	0.03	0.00	0.00	0.00
16.600–16.700	0.01	0.01	0.01	0.01
16.700–16.800	0.03	0.00	0.00	0.00
16.800–16.900	0.05	0.06	0.08	0.05

**Table A14:** Estimates of the railway track condition—relative *D_6_*, object No. R_161_1._

Railway Track Baseline Sections	2019	2020	2021	2022
4.400–4.500	-	-	0.00	0.00
4.500–4.600	-	-	0.00	0.00
4.600–4.700	-	-	0.00	0.00
4.700–4.800	-	-	0.00	0.00
4.800–4.900	-	-	0.11	0.07
9.500–9.600	-	-	0.00	0.00
9.600–9.700	-	-	0.00	0.00
9.700–9.800	-	-	0.00	0.00
9.800–9.900	-	-	0.00	0.00
9.900–10.000	-	-	0.00	0.00
10.000–10.100	-	-	0.00	0.00
10.100–10.200	-	-	0.00	0.00
10.200–10.300	-	-	0.00	0.00

**Table A15:** Estimates of the railway track condition—relative *D_6_*, object No. R_161_2._

Railway Track Baseline Sections	2019	2020	2021	2022
4.400–4.500	-	-	0.00	0.00
4.500–4.600	-	-	0.00	0.00
4.600–4.700	-	-	0.00	0.00
4.700–4.800	-	-	0.00	0.00
4.800–4.900	-	-	0.01	0.00
9.500–9.600	-	-	0.00	0.00
9.600–9.700	-	-	0.00	0.00
9.700–9.800	-	-	0.00	0.00
9.800–9.900	-	-	0.00	0.00
9.900–10.000	-	-	0.00	0.00
10.000–10.100	-	-	0.00	0.00
10.100–10.200	-	-	0.00	0.00
10.200–10.300	-	-	0.00	0.00
